# Extracellular Vesicle-Derived microRNAs: Novel Non-Invasive Biomarkers for Gastrointestinal Malignancies

**DOI:** 10.3390/ijms27010010

**Published:** 2025-12-19

**Authors:** Daniela Nardozi, Valeria Lucarini, Valentina Angiolini, Nicole Feverati, Monica Benvenuto, Chiara Focaccetti, Letizia Del Conte, Olga Buccitti, Camilla Palumbo, Loredana Cifaldi, Elisabetta Ferretti, Roberto Bei, Laura Masuelli

**Affiliations:** 1 Department of Experimental Medicine, University of Rome “Sapienza”, Viale Regina Elena 324, 00161 Rome, Italy; daniela.nardozi@uniroma1.it (D.N.); valentina.angiolini@uniroma1.it (V.A.); nicole.feverati@uniroma1.it (N.F.); letizia.delconte@uniroma1.it (L.D.C.); elisabetta.ferretti@uniroma1.it (E.F.); 2 Department of Life Science, Health, and Health Professions, Link Campus University, 00165 Rome, Italy; v.lucarini@unilink.it; 3 Department of Clinical Sciences and Translational Medicine, University of Rome “Tor Vergata”, Via Montpellier 1, 00133 Rome, Italy; monica.benvenuto@uniroma2.it (M.B.); chiara.focaccetti@uniroma2.it (C.F.); b.olga999@gmail.com (O.B.); plmcll00@uniroma2.it (C.P.); cifaldi@med.uniroma2.it (L.C.); bei@med.uniroma2.it (R.B.)

**Keywords:** gastrointestinal tumors, miRNA, biomarker, tumor microenvironment, tumor progression, metastasis

## Abstract

Gastrointestinal (GI) cancers represent a heterogeneous group of malignant neoplasms arising from the digestive tract, including gastric, colorectal, hepatic, pancreatic, and biliary cancers. These tumors represent a major public health challenge due to their aggressive nature and poor prognosis. Although significant progress has been made in diagnostic imaging, endoscopy, and multimodal therapies, early detection remains difficult. Conventional serum biomarkers often lack sufficient sensitivity and specificity for reliable diagnosis, prompting a growing interest in identifying novel, minimally invasive biomarkers. In this context, liquid biopsy is emerging as a revolutionary tool in oncology. Among its components, extracellular vesicles (EVs) have gained increasing attention because they carry a wide range of molecular cargoes that reflect the biological state of their tumor of origin. In particular, EV-associated microRNAs (miRNAs) hold great promise as biomarkers for early cancer detection, real-time monitoring of disease progression, and assessment of therapeutic response. This review discusses the diagnostic and prognostic potential of EVs as novel biomarkers in GI cancers, emphasizing EV-contained miRNAs as a key resource for the development of personalized and precision medicine strategies.

## 1. Introduction

Gastrointestinal (GI) cancers are a group of neoplasms that arise in the GI tract and include malignant diseases of the stomach, intestines, liver, pancreas, and other parts of the digestive apparatus [1,2]. Given their highly aggressive potential and high mortality rates, corresponding to more than 35% of all cancer deaths, GI tumors represent a significant health problem [2,3,4]. For this reason, considerable efforts in recent years have focused on improving therapeutic outcomes through early diagnosis and multimodality treatment often involving a combination of surgery, chemotherapy, and radiation therapy [5]. Advances in imaging and endoscopic techniques have improved the ability to effectively diagnose and manage these tumors [6], and biomarkers are playing an increasingly important role. Glycoproteins, such as carcinoembryonic antigen (CEA), cancer antigen 19-9 (CA19-9), carbohydrate antigen 15–3 (CA 15–3), and carbohydrate antigen 12–5 (CA 12–5), are classical circulating biomarkers for GI tumors [7]. However, these biomarkers often lack the sensitivity and specificity requirements that would be needed for a reliable GI tumor diagnosis. Therefore, the identification of new sensitive and specific biomarkers is of crucial importance. In this context, the “liquid biopsy” concept has emerged as a transformative tool in the past two decades [8]. Liquid biopsy represents a key advancement in oncology, as it offers a minimally invasive and safely repeatable method to detect tumors and monitor their progression over time. Unlike traditional tissue biopsies, which require surgical procedures to obtain tumor samples, liquid biopsies consist of samples of blood or other body fluids that can be analyzed for their content of tumor-derived components. This approach enables early detection of tumors, continuous monitoring of disease progression, and dynamic assessment of therapeutic responses [8,9,10,11].

The content of a liquid biopsy mainly includes circulating tumor DNA (ctDNA), circulating tumor cells (CTCs), extracellular vesicles (EVs), circulating cell-free RNA (cfRNA), including small RNAs/microRNAs (miRNAs), and various proteins and metabolites released from the tumor into the bloodstream [12]. The ctDNA fragments carry genetic and epigenetic signatures that reflect the mutational landscape of the tumor, and can provide information on both genetic alterations and resistance mechanisms. A similar role is played by cfRNAs [13,14]. CTCs, although rare, can be analyzed to obtain data on the tumor’s phenotypic and genotypic features. Among the tumor-derived components that can be found in liquid biopsies, EVs have attracted considerable attention in recent years. This is because their molecular cargo, derived from the cell of origin, simultaneously contains multiple biological analytes including nucleic acids, proteins, lipids and carbohydrates [13,14,15]. Therefore, the analysis of the molecules contained in liquid biopsies’ EVs can be quite helpful for the early detection of different types of cancer.

In this review, we highlight the central role of the liquid biopsy approach in monitoring the progression of GI tumors, with a focus on EVs and their cargo as emerging biomarkers with enhanced sensitivity, able to provide useful data for patient-tailored therapeutic strategies against GI tumors.

## 2. Extracellular Vesicles

EVs are small lipid membrane-enclosed structures that are released into the extracellular environment by almost every cell type and that can be easily detected in various body fluids, including blood, lymph, saliva, urine, cerebrospinal fluids, breast milk, tears and ascitic or pleural effusions [16,17,18].

In 1983, Pan and Johnstone were among the pioneers in discovering the characteristics of EVs during their studies of the maturation of reticulocytes into erythrocytes [19]. EVs were originally believed to be cellular debris, but later it was highlighted their important role in intercellular communication [20].

EVs can be categorized into three main types based on their biogenesis and size range: exosomes, microvesicles (MVs) and apoptotic bodies [21]. Exosomes are small membrane vesicles ranging from 30 to 150 nm in diameter, resulting from the exocytosis of intracellular multivesicular bodies [22,23]. MVs are larger membrane vesicles with diameters ranging from 150 to 1000 nm, which bud off from the plasma membrane [21,22]. It is noteworthy that every cell can simultaneously release both exosomes and MVs. Apoptotic bodies typically exhibit larger dimensions, ranging from 50 to 5000 nm, are formed during programmed cell death, and are identified by the inclusion of organelles within the vesicles [22,24].

Recently, attention has been focused on a population of cancer-derived EVs, known as “oncosomes”, that originate from the plasma membrane and present an unusually larger size (1–10 μm in diameter) compared to other EV types. Due to their atypical dimensions, oncosomes are thought to have distinctive properties in vivo, offering new potential tools for tumor profiling approaches [25].

In cancer, it is well known that EVs have a fundamental role in intercellular communication as vehicles that deliver bioactive cargo to recipient cells, thereby modulating cellular functions in both paracrine and autocrine manners [21,25,26,27].

By regulating cellular processes, such as proliferation, differentiation and apoptosis, EVs participate in maintaining tissue homeostasis, as well as in tissue repair and regeneration after injury [28,29]. Moreover, EVs contribute to angiogenesis and tissue healing by delivering pro-angiogenic factors to endothelial cells [30].

EVs are involved in immune regulation with non-univocal effects on immune cells: they can either (i) promote immune responses, as reported infectious processes, or (ii) suppress allergic, autoimmune and antitumor immune reactions, as it has been reported to occur in tolerance induction or immune evasion by cancer cells [31].

Overall, EVs represent a versatile mechanism for intercellular communication and exert significant effects on both physiological and pathological processes. Understanding their functions and mechanisms of action is a rapidly evolving area of research with implications for diagnostics, therapeutics, and regenerative medicine. Among these implications, the rapid isolation and characterization of liquid biopsy-derived EVs is becoming increasingly crucial in cancer diagnosis, prognosis, and therapy [32].

In addition, EVs have a distinctive biophysical property: exceptional stability in extracellular environments [33]. Their lipid bilayer encapsulation protects cargo from enzymatic degradation by RNases, DNases, and proteases, as well as mechanical stress, pH fluctuations, and other extracellular insults present in biological fluids. This protection preserves proteins, lipids, and nucleic acids, especially miRNAs, maintaining integrity during circulation and intercellular transfer [34,35]. Furthermore, their stability supports long-distance signaling and their reliability as liquid biopsy biomarkers [35,36].

For this reason, in the last decade, researchers have developed numerous methods for the isolation of EVs, including ultracentrifugation, size exclusion chromatography, immunoaffinity capture, polymeric precipitation and microfluidics methods [32,37]. These approaches can differentially influence EV size distribution and molecular cargo, potentially introducing methodological bias in biomarker detection and quantification. Therefore, in the context of EV isolation protocols, careful optimization, standardization, and cross-validation are essential to ensure the accuracy, reproducibility, and translational applicability of EV-miRNA profiling in clinical settings [38,39].

Importantly, many clinical trials involve cancer-derived EVs as a biomarker for cancer prediction, stage, prognosis, diagnosis and therapeutic treatment response [18,36], considering that tumor cells exhibit an increased and dysregulated release of EVs, caused by the activation of oncogenic signaling pathways or altered Rab GTPase activity (e.g., Rab27a/b) [40,41]. This determines a higher concentration of circulating EVs, which correlates with tumor burden and stage of disease.

### 2.1. Biogenesis of Extracellular Vesicles

EV biogenesis involves complex cellular processes and requires an accurate regulation of multiple intracellular trafficking pathways, shaping the composition of newly formed vesicles and influencing their production. As we previously mentioned, there are three main types of EVs, exosomes, MVs, and apoptotic bodies, and each has a distinct biogenesis pathway [21,22,24,28,42,43].

Even if exosomes and MVs are generated in different cellular compartments, their biogenesis involves common intracellular mechanisms and sorting machineries [42,44].

Exosomes originate from the endosomal system within the cell, starting with cargo endocytosis and subsequent formation of endocytic vesicles in specialized regions of the plasma membrane. These vesicles detach from the plasma membrane and fuse with the early endosomes, which then mature into late endosomes, also known as multivesicular bodies [28]. Late endosomes can undertake two different fates: typically, it fuses with the lysosome for degradation, but in some cases, they can fuse with the plasma membrane, releasing the intraluminal vesicles (ILVs), which are formed by endosomal membrane invagination in the organelle lumen, into the extracellular space as exosomes [17,21,28,45].

In contrast to exosomes’ biogenesis, MVs and apoptotic bodies originate by budding from the plasma membrane. MVs formation arises from the dynamic interplay between the redistribution of membrane phospholipids and the rearrangement of cytoskeletal proteins. The budding process involves the contraction of cytoskeletal structures through actin-myosin interactions, leading to the release of MVs into the extracellular space [22,42,43]. Apoptotic bodies are produced during programmed cell death (apoptosis). During apoptosis, cells undergo multiple structural changes, including DNA fragmentation, chromatin condensation, and membrane blebbing. These membrane-bound vesicles contain cellular organelles and fragmented DNA and are subsequently engulfed and cleared by phagocytic cells [24,42,43].

The biogenesis of EVs is regulated by various cellular processes and signaling pathways, including those involved in membrane trafficking, cytoskeletal dynamics, and cellular stress responses [42,43]. Understanding the mechanisms underlying EV biogenesis is crucial for elucidating their roles in physiological and pathological processes, including intercellular communication, immune regulation, and disease progression [42,43].

### 2.2. Uptake of Extracellular Vesicles

The growing interest in EVs is linked to their potential to induce changes in the physiology of recipient cells. The way EVs are captured by the recipient cells varies depending on the EV surface components and size [46].

There are several mechanisms by which EVs can communicate with recipient cells. Specific interactions between EV surface molecules (such as ligands) and receptors on recipient cells can facilitate EV uptake and activate downstream signal transduction pathways. For example, cellular integrins interact with ICAMs expressed on the surface of the exosome, or with integrins present on the exosome, through components of the extracellular matrix [47].

EVs can be internalized by recipient cells through various endocytic pathways such as clathrin-dependent or independent endocytosis, caveolin-mediated endocytosis, or micropinocytosis. In more detail, EVs can fuse directly with the plasma membrane of recipient cells by interacting with specific proteins and lipids and reach the lysosome, serving as a reservoir of metabolic materials; alternatively, they can avoid the fusion with the late endosome and release their content into the cytoplasm of the recipient cell, thereby influencing various cellular processes [48]. The docking between EVs and the target cell is facilitated by interactions involving tetraspanins, integrins, and other adhesion molecules present on both the EV surface and recipient cell membrane [46,49].

### 2.3. Cargo Selection

EVs can contain various molecules, including lipids, proteins, and nucleic acids (including DNA and the entire repertoire of RNAs). This content can vary depending on various factors such as their biogenesis, cell of origin, and the physiological or pathological conditions under which they are formed [50,51].

Since the specific cargo loaded into EVs can vary significantly from one EV to another and between EVs derived from different cell types, researchers have conducted extensive studies aimed at characterizing EVs’ contents and understanding the complexities of cargo selection mechanisms [50]. Moreover, understanding the mechanisms behind cargo sorting in EVs is crucial for devising strategies to utilize EVs for diagnostic and therapeutic applications.

EV cargo selection is a complex, dynamic, and highly regulated process that involves various mechanisms to selectively package specific biomolecules. The physiological state of cells can influence EV cargo selection. For example, cells undergoing apoptosis or cellular stress may release EVs containing specific biomolecules associated with these processes. Similarly, cell type, developmental stage, and changes in the microenvironment can also influence EV cargo composition [52].

During exosomes biogenesis, the Endosomal Sorting Complex Required for Transport (ESCRT) machinery allows, in fact, specific proteins and nucleic acids to be selectively incorporated into ILVs, contributing to the cargo of exosomes [53]. Furthermore, tetraspanins (such as CD9, CD63, and CD81) and lipid rafts, which are cholesterol-rich microdomains in the plasma membrane, play a crucial role in EVs biogenesis and cargo sorting because they can interact with specific cargo molecules and facilitate their packaging into EVs [54].

The selective packaging of RNAs into EVs is regulated by specific sorting machineries that involve RNA-binding proteins (RBPs) and their associated partners. RBPs, like heterogeneous nuclear ribonucleoproteins (hnRNPs) and members of the Argonaute (Ago) protein family, can bind to specific RNA sequences, target them to the sites of EV generation and protect them from degradation [55].

### 2.4. EVs as microRNA Carrier

The therapeutic potential of EVs is attracting increasing interest. Indeed, EVs could be transformed into sophisticated carriers able to deliver therapeutic proteins, RNA molecules, and drugs into specific cells [42]. The ability of these natural vectors to perform cellular targeting, to easily penetrate cell membranes and cell organelles, their special physico-chemical characteristics, and their ability to circulate without being detected by the immune system are very attractive features [56,57].

In recent years, particular attention has been paid to miRNAs, short endogenous single-stranded noncoding RNA sequences of about 17–23 nucleotides that can regulate gene expression by binding to target messenger RNAs (mRNAs), leading to their degradation or post-transcriptional changes [58].

Conventionally, there are standardized guidelines for the identification and annotation of miRNAs in order to reliably distinguish miRNAs from other RNAs, such as small interfering RNAs [59].

It has been shown that all types of EVs can transport miRNAs, which modulate gene expression in target cells in both normal and pathological conditions. Although the mechanisms by which miRNAs are sorted into EVs or retained within the cell of origin are still largely unknown [57], it has been demonstrated that miRNAs contain sorting sequences, EXOmotifs and CELLmotifs, that determine their secretion into EVs or their retention, respectively [60]. This selective loading of miRNAs into EVs is mediated by RBP, such as hnRNPA2B1, Ago2 and YBX1, which recognize specific sequence motifs in the miRNAs, guiding their inclusion into exosomes and ensuring that the EV miRNA signature reflects the physiological or pathological state of the producing cell, including malignant transformation [61,62,63]. Furthermore, different cell types preferentially use specific sorting sequences, so that the EVs’ miRNA profiles could reveal both the originating cell type and the cell’s functional status [60].

Moreover, EVs serve as carriers that protect miRNAs from degradation by extracellular RNases. This protection ensures the stability and integrity of miRNAs during their transfer between cells and through various biological fluids [60,64,65], offering a clear mechanistic advantage compared to free miRNAs, giving EV-miRNAs superior stability in circulation and making them highly suitable for detection in liquid biopsies [66].

The transfer of miRNAs to recipient cells via EVs can have multiple functional implications. For example, the EV-mediated transfer of tumor-suppressive miRNAs from normal cells to cancer cells can inhibit tumor growth and metastasis. Conversely, cancer-derived EVs can transfer oncogenic miRNAs to neighboring cells, promoting tumor progression and metastasis [67,68]. In GI cancers, EV-derived miRNAs have been reported to regulate critical processes such as epithelial–mesenchymal transition (EMT), angiogenesis, chemoresistance, tumor-associated inflammation and the formation of the pre-metastatic niche [69]. Since EV release increases with tumor aggressiveness and metabolic stress, the abundance of specific EV-miRNAs frequently correlates with tumor stage, lymph node involvement, metastasis and prognosis [61,62].

However, the EV isolation protocol previously mentioned can differentially influence EV size distribution and miRNA cargo, potentially introducing methodological bias in biomarker detection and quantification. Therefore, in the context of EV isolation protocols, careful optimization, standardization, and cross-validation are essential to ensure the accuracy, reproducibility, and translational applicability of EV-miRNA profiling in a clinical perspective [38,39].

## 3. Role of EVs as Biomarkers in Gastrointestinal Cancer Progression

Recent studies have highlighted the pivotal role of EVs in the development and progression of solid tumors, due to their important role as well in communication between tumors and the microenvironment. In fact, tumor cells can modify the behavior of both nearby and distant normal cells by transferring the cargo of EVs, which in turn supports tumor growth and metastasis [15,70,71,72].

In GI tumors, EVs can contribute to tumor growth, immune evasion, and metastasis by modulating the behavior of surrounding cells and creating a microenvironment favorable to cancer progression [15]. Furthermore, EVs have emerged as promising tools for both early detection and targeted therapy, opening new avenues for improving the diagnosis and treatment of GI cancers [15,73,74].

The detection of mutated DNA within the EVs’ cargo may indicate the presence of specific cancer types. For example, KRAS gene mutations in EVs serve as a potential biomarker for pancreatic or colon cancers [75,76]. However, the overall amount of DNA in EVs released from cancer cells is relatively low, and some mutations can also be observed in EVs derived from healthy subjects. Therefore, the sensitivity and specificity of such mutated DNA may not be sufficient for clinical applications.

As well, noncoding RNAs, including miRNA, circular RNA (circRNA), long non-coding RNA (lncRNA) and Piwi-interacting RNA (piRNA), are enriched in EVs isolated from cancer patients, resulting in a high impact on disease progression, through the promotion of neoangiogenesis and metastasis formation [70,77]. Given that their high levels in EVs allow an easy detection and effective amplification by PCR, they represent more reliable diagnostic candidates [4,74,78]. Additionally, EVs can carry tumor-specific proteins, both encapsulated within their lumen and expressed on their surface, providing further opportunities for early cancer detection [79].

It has been thoroughly investigated that compared to free circulating and tissue miRNAs, those from EVs are preferable biomarkers due to their stability against degradation and enhanced specificity for the source cell [80,81]. As a matter of fact, tissue miRNAs directly reflect specific tissues but require invasive collection methods, whereas circulating miRNAs tend to be unstable and are often diluted by miRNAs from other cell types, such as platelets and red blood cells. In contrast, EV-derived miRNAs represent a non-invasive choice that preserves both stability and specificity, positioning them as a more promising diagnostic and prognostic biomarker [80,81].

EVs derived from GI tumor cells (GI-EVs) promote angiogenesis by delivering pro-angiogenic factors such as vascular endothelial growth factor (VEGF) and miRNAs that modulate endothelial cell behavior. Additionally, GI-EVs contribute to immune evasion through the transfer of immunosuppressive molecules like PD-L1 and TGF-β and, yet, may confer drug resistance by polarizing macrophages [4,82,83].

A crucial aspect of GI-EVs’ function is their role in metastasis. Tumor-derived EVs can prime distant organs for metastasis by conditioning the pre-metastatic niche and altering the local microenvironment to support the survival of metastatic cancer cells and their colonization at the site [84,85]. For example, pancreatic cancer-derived EVs have been found to carry integrins that direct them to specific organs, where they interact with resident cells to remodel the extracellular matrix and promote the formation of a metastatic niche [86]. Specific miRNA signatures in EVs have been associated with colorectal cancer (CRC) stages and therapeutic outcomes [87].

In summary, EVs are central to the pathophysiology of GI tumors, influencing angiogenesis, immune evasion, metastasis, and offering promise as diagnostic and therapeutic tools. Understanding their complex roles and mechanisms may unlock new possibilities for fighting GI cancers.

Since traditional diagnostic methods, such as endoscopy and biopsy, are invasive and often detect cancer at advanced stages, the use of EVs as diagnostic biomarkers is a rapidly evolving field and offers a non-invasive alternative.

### 3.1. Gastric Cancer

Gastric cancer (GC) originates from the stomach lining. It is the fifth most common cancer in the world and the third leading cause of cancer death [88]. Despite advances in medical treatments and even though nowadays there are a variety of treatment strategies for GC, there has been no significant improvement in the outcome of patients with advanced-stage disease, underscoring the need for further research and more effective screening methods [89,90].

miRNA-containing EVs play a key role in GC progression by promoting cell proliferation through the activation of PI3K/Akt and mitogen-activated protein kinase (MAPK) signaling pathways [91,92]. In fact, they are released by GC cells and significantly influence tumor behavior and the tumor microenvironment (TME) by promoting tumor growth, metastasis, and resistance to chemotherapy through the silencing of tumor suppressor genes and the activation of oncogenic pathways in recipient cells [93]. Specifically, miR-149-5p affects the expression level of AKT1 in several GC cell lines, thus promoting tumor growth [94]. It has also been shown that GC cell lines resistant to paclitaxel, one of the main chemotherapeutic agents used in GC, transfer miR-155p through EVs to sensitive cells, thereby inducing chemoresistance in the latter [95]. Other studies conducted on GC cell cultures underline the role of EV, through the upregulation of miR23a, in promoting angiogenesis via the repression of PTEN [96]. EVs also play an important role in the interactions between GC cells and immune cells within the TME. miR-519a-3p has a critical role in mediating the crosstalk between primary GC cells and intrahepatic macrophages through MAPK/ERK pathways stimulation, thus promoting angiogenesis and metastasis, and represents a potential therapeutic target for GC [97]. Likewise, exosomal miR-151-3p causes M2-like macrophage polarization, favoring tumor progression [98]. In turn, miR-21 contained in EVs from M2-polarized macrophages can promote chemoresistance by supporting cancer cell survival and proliferation [83,99].

Several studies report the pivotal role of miRNAs contained in EVs as diagnostic and prognostic biomarkers. miR-19b and miR-106a are associated with GC progression and metastasis. In more detail, studies conducted on liquid biopsies of GC patients revealed that exosome-derived miR-106a directly interacts with Smad7, inducing peritoneal metastasis in GC, while miR-19b, isolated from the serum of GC patients, exhibits an oncogenic role and is able to discriminate between healthy controls and GC patients. Accordingly, these miRNAs have promising characteristics as potential biomarkers [100,101]. EV-derived miRNAs that could be used as biomarkers of prognosis or drug resistance include the exosome-derived miR-451, which induces Th17 differentiation by increasing mammalian target of rapamycin (mTOR) activity, and miR-493, whose expression correlates with paclitaxel resistance in human GC cell lines [102,103].

The principal EV-derived miRNAs used as biomarkers in GC are listed in Table 1.

### 3.2. Colorectal Cancer

CRC is one of the most common cancers worldwide, for which treatment options usually include a combination of surgery, chemotherapy, radiation, and targeted therapies, depending on the stage and specific location of the tumor [3]. Even in CRC, EVs play a crucial role in tumor progression and TME modulation, and can serve as biomarkers for diagnosis, prognosis, and therapeutic response. For instance, EVs’ release results in increased tumor invasiveness by transferring tumor promoters such as mutant KRAS, Epidermal Growth Factor Receptor (EGFR) and integrins to tumor cells with wild-type KRAS [108].

Similarly, miRNAs contained in EVs have a key role, with some promoting tumor growth, invasion, and metastasis by targeting tumor suppressor genes or modulating signaling pathways [109]. For example, EVs carrying miR-19a can promote CRC progression by suppressing the expression of TIA1, a tumor suppressor gene [109]. Conversely, EVs containing miR-145 can inhibit CRC progression and metastatic spread by targeting genes involved in cell proliferation and migration [110,111]. Exosome-derived miR-183-5p, highly expressed in CRC cells, induces cell proliferation and migration by targeting the transcription factor forkhead box O1 (FOXO1) [112]. EV-derived miR-92a can enhance angiogenesis by targeting and downregulating integrin α5 [113,114,115]. Exosome-derived miRNAs, such as miR-106b, miR-146a and miR-155-5p, are able to trigger the EMT and promote metastasis through different mechanisms, including macrophage polarization, activation of the PI3Kγ/Akt/mTOR pathway, or targeting of the suppressor of cytokine signaling 1 (SOCS1) [116,117]. Other exosomal miRNAs involved in tumor progression and metastasis induction are miR-203a and miR-934, which target PTEN and induce macrophage polarization [118,119]. Furthermore, it has been reported that EVs derived from cancer-associated fibroblasts (CAFs) transport lncRNA19 to CRC cells, inducing stemness properties and drug resistance through the activation of Wnt and β-catenin pathways [120]. Finally, some miRNAs can influence the immune response by modulating the activity of immune cells. For example, miR-21 contained in EVs can suppress the immune response, promoting tumor immune evasion [121].

EV-derived miRNAs can also be used as biomarkers of CRC progression through the analysis of liquid biopsies; in fact, they can serve as diagnostic biomarkers, indicating the presence of CRC in the early stages, and provide prognostic information. For example, miR-21 and miR-23a can be detected in plasma-derived EVs from CRC patients, showing high sensitivity and specificity [114]. Indeed, miR-21 plasma levels could be used as a diagnostic biomarker since it is differentially expressed between CRC patients and healthy donors, likewise miR-23a and miR-1246 [114,122].

Furthermore, miR-21 carried by EVs has been identified as a potential therapeutic biomarker, as it contributes to 5-fluorouracil resistance by targeting the PTEN/PI3K/AKT pathway, which controls genes involved in drug metabolism, ultimately promoting chemoresistance [123,124].

MiR-92a could also represent a promising biomarker, since it has been demonstrated that its expression is high in the serum of stage I and stage II CRC patients [114,125]. Elevated levels of some miRNAs, such as miR-200c, are associated with poor prognosis and advanced stages of the disease [114]. High levels of serum EVs containing miR-181a-5p have been positively correlated with liver metastasis in CRC patients [126].

In liquid biopsies of CRC patients, EV-derived miR-320c expression is linked to lower overall survival and metastatic niche formation, so that it can be used as a biomarker for therapeutic monitoring [127].

Table 2 lists some of the principal EV-derived miRNAs investigated as biomarkers in CRC.

### 3.3. Pancreatic Cancer

Pancreatic cancer (PC) is a highly aggressive and often fatal disease [132]. Due to its nonspecific early symptoms, such as abdominal pain and weight loss, PC is frequently diagnosed at an advanced stage, making effective treatment challenging. Despite advances in medical research, the prognosis remains poor, with a five-year survival rate of around 10%, underscoring the critical need for early detection and improved therapeutic strategies [133]. Pancreatic ductal adenocarcinoma (PDAC) is the most common PC variant, characterized by a very low survival rate due to the high incidence of metastasis and late diagnosis [134].

In vitro studies have shown that EVs play an important role in PDAC chemoresistance. For instance, EVs released from gemcitabine-exposed CAFs increase the proliferation and survival of chemoresistant PDAC cell lines through the regulation of the transcription factors SNAIL and miR-146a [135,136]. Exosomal miR-106b, released from CAFs after TP53INP1-targeted treatment, also induces gemcitabine resistance [137]. In addition, EVs from gemcitabine-resistant PDAC stem cells can transfer drug resistance to gemcitabine-sensitive PDAC cells by carrying miR-210, which targets the mTOR signaling pathway [73].

Tumor progression toward an invasive phenotype through the EMT is also promoted by PDAC-derived EVs. In this regard, it has been reported that tumor EVs containing tenascin C induce Wnt/β-catenin signaling, EMT, and tumor progression [86,138].

miR-34a, which typically acts as a tumor suppressor, is downregulated in PC [139]. It can inhibit cancer cell proliferation, migration, and invasion by targeting several genes involved in EMT. Loss of miR-34a contributes to uncontrolled tumor growth and metastatic potential [139,140]. The delivery of a miR-34a coated with exosomes (exomiR-34a) to PC cells has been shown to significantly inhibit their growth [140]. Conversely, miR-1246 is known to be upregulated in PC exosomes and has been linked to EMT and chemoresistance [135]. By targeting and downregulating tumor suppressor genes, miR-1246 supports a more aggressive cancer phenotype.

As mentioned before, miRNAs can modulate the immune landscape, supporting immune evasion by cancer cells. For instance, EV-derived miR-203 can reduce the anti-tumor immune response by downregulating SOCS3 and promoting immunosuppressive environments [141]. miRNAs can also influence stromal cells within the TME. EVs containing miR-1246 can activate pancreatic stellate cells, thereby promoting desmoplasia and facilitating tumor growth [86].

Numerous studies have demonstrated how EVs from PC patients contain specific miRNAs that can serve as diagnostic and prognostic markers. For instance, elevated levels of miR-19b, miR-21, miR-27b, miR-210 and miR-221 are frequently found in EVs from PC patients compared to healthy donors and are associated with tumor progression, metastasis, and poor prognosis [138,142,143,144,145]. Moreover, exosome-derived miR-21, as well as miR-10b, miR-181 and miR-121, are usually upregulated in PDAC patients compared to healthy donors [142]. Elevated levels of miR-21 in EVs have also been correlated with poor prognosis and advanced disease, highlighting its potential as a prognostic biomarker [142]. Exosome-derived miR-19b is regarded as a promising diagnostic marker, allowing to discriminate between patients with PC and patients with chronic pancreatitis or healthy controls [146]. miR-301a-3p carried by exosomes promotes migration and invasion of PC cells by targeting PTEN and activating the PI3K signaling pathway, so that it could be a valid diagnostic and prognostic biomarker [147,148]. In addition, miR-221 targets tumor suppressor genes such as PTEN and PDCD4, thereby activating pathways that lead to increased tumor growth and resistance to apoptosis [138]. On the contrary, EVs containing miR-146a can suppress PC cell invasion and metastasis by downregulating the EGFR/ERK pathway [149].

The principal EV-derived miRNAs used as biomarkers in PC and PDAC are listed in Table 3.

### 3.4. Hepatocarcinoma

Hepatocarcinoma (HCC) is the most common primary malignant tumor of the liver and accounts for about 75–85% of all liver cancers [151]. It typically arises in the context of chronic liver disease, particularly cirrhosis caused by hepatitis B or C infection, chronic alcohol abuse, or nonalcoholic fatty liver disease [151]. HCC has a poor prognosis because of its aggressive nature and late presentation in many patients. Treatment options, which are limited and differ according to the stage of the disease, include surgical resection, liver transplantation, and systemic therapies, such as targeted treatments and immunotherapy [152]. Early detection and surveillance in high-risk populations are critical to improving outcomes. EVs have a significant role in HCC progression and are involved in processes such as cell proliferation, EMT and immune response modulation. High levels of miR-21 in EVs have been correlated with tumor progression, by targeting PTEN and AKT-signaling, and with poor prognosis in HCC patients [153,154,155]. Moreover, exosome-derived miR-21 isolated from HCC cell lines contributes to tumor progression by promoting angiogenesis and EMT [155,156]. In addition, EV-derived miR-3129, isolated from patients’ plasma, enhances HCC growth and promotes metastasis formation [157]. On the other hand, the downregulation of exosomal miR-200b-3p in HCC cells promotes angiogenesis through the increased expression of endothelial ERG [158].

Different studies have identified specific miRNAs in serum-derived EVs that are associated with HCC, such as miR-21, miR-122, and miR-192, often dysregulated in cancer cells [82,153,159]. Among these miRNAs, miR-122 is a liver-specific miRNA that has been found to be significantly reduced in EVs from HCC patients, reflecting its role in liver homeostasis and its potential as a diagnostic marker [160,161]. It has also been demonstrated that EV-derived miR-210 targets SMAD4 and STAT6, inducing angiogenesis and tumor progression [162]. Other EV-derived miRNAs that could be used as diagnostic biomarkers due to their upregulation in HCC patients’ serum are miR-15b, miR-16, miR-150 and miR-26a [163].

It has been reported that exosomal miR-106a expression, increased in HCC patients’ serum, correlates with a poor prognosis. In fact, miR-106a regulates the MAPK and c-Jun N-terminal kinase (JNK) pathways, thus supporting tumor progression [164].

miRNAs of the miR-199 family are instead considered diagnostic biomarkers downregulated in HCC patients. Notably, the low expression of miR-199a/b-3p, the third most abundantly expressed miRNA in the liver, is associated with poor overall survival. miR-199a plays a pivotal role in controlling tumor aggressiveness by influencing E-cadherin regulation. In addition, overexpression of miR-199 family significantly suppresses HCC cell proliferation, migration, and invasion by inhibiting the Regulators of G-protein signaling (RGS) pathway. These findings suggest that restoring normal levels of miR-199a/b-3p could serve as a potential therapeutic strategy for HCC, able to target multiple pathways involved in HCC growth and aggressiveness [153].

Another miRNA that can act as a tumor suppressor in HCC is miR-214, which prevents tumor growth via the inhibition of β-catenin, a key player in cancer progression [165]. Exosomal miR-214 also inhibits tumor cell proliferation, migration, and metabolic activity by targeting Pyruvate Dehydrogenase Kinase Isoform 2 (PDK2) and PHD Finger Protein 6 (PHF6) in HCC cell cultures. In addition, miR-214 negatively regulates PVT1, an lncRNA linked to HCC development. Elevated levels of miR-214 have been associated with reduced PVT1 expression both in in vivo and in vitro studies, and PVT1 silencing through miR-214 or siRNA significantly reduced HCC cell viability and invasiveness [153,166].

A recent study by Boonkaew et al. showed that plasma EV-miRNAs could effectively discriminate HCC patients from healthy ones. In particular, miR-223 expression levels were significantly higher in patients with HCC compared with healthy controls [167]. Hence, it may potentially serve as a biomarker for HCC progression and patient outcomes [153,168].

The principal EV-derived miRNAs used as biomarkers in HCC are listed in Table 4.

### 3.5. Cholangiocarcinoma

Cholangiocarcinoma (CCA) is an aggressive and heterogeneous malignancy originating from the bile duct epithelium [169]. It is associated with an adverse prognosis and high mortality and, due to its increasing global incidence, there is an urgent need for effective early detection and treatment strategies [169]. EVs, including exosomes and MVs, determine and influence different cellular processes in CCA, such as proliferation, invasion, and metastasis [170], and their contents have recently emerged as ideal biomarkers for liquid biopsy-based diagnosis and prognosis of CCA [9,171].

In this regard, specific miRNAs within exosomes have been identified as key players in CCA tumorigenesis and metastasis. miR-21 is frequently upregulated in many GI cancers, including CCA, and is known for its role in promoting cell proliferation, invasion, and survival [172]. Furthermore, miRNAs carried by EVs from CCA patients have shown promise in distinguishing between malignant and benign biliary conditions. High-throughput analysis, for example, highlighted that miR-9, a well-known marker of biliary tract cancer [173], was increased in CCA patients. Moreover, EV-associated miR-21 and miR-222 are frequently upregulated in CCA and correlate with tumor progression and poor prognosis. These miRNAs were, in fact, overexpressed in either plasma or serum of CCA patients compared to healthy donors [174,175]. In addition, miR-221 promotes cell cycle progression and inhibits apoptosis by targeting and downregulating cell cycle inhibitors such as p27Kip1 and pro-apoptotic proteins [176]. Its overexpression is often correlated with poor prognosis and advanced disease stages. Members of the miR-200 family, including miR-200c and miR-141, are involved in regulating EMT in CCA by targeting ZEB1 and ZEB2, which are transcriptional repressors of E-cadherin [177]. Dysregulation of these miRNAs in exosomes can lead to enhanced metastatic capabilities of CCA cells [178]. These miRNAs within EVs not only reflect the biological behavior of CCA cells, but also actively contribute to modifying the TME and promoting disease progression.

Lastly, the upregulation of EV-derived miRNAs, such as miR-34c, miR-30e and miR-195, leads to CCA development and progression by targeting Snail and inducing EMT; for this reason, they could be used as potential diagnostic biomarkers [179,180,181]. Overall, their detection in bodily fluids like blood and bile offers a promising approach for non-invasive diagnostics and monitoring, while their specific pathways provide targets for potential therapeutic interventions. The principal EV-derived miRNAs used as biomarkers in CCA are listed in Table 5.

**Figure 1:**
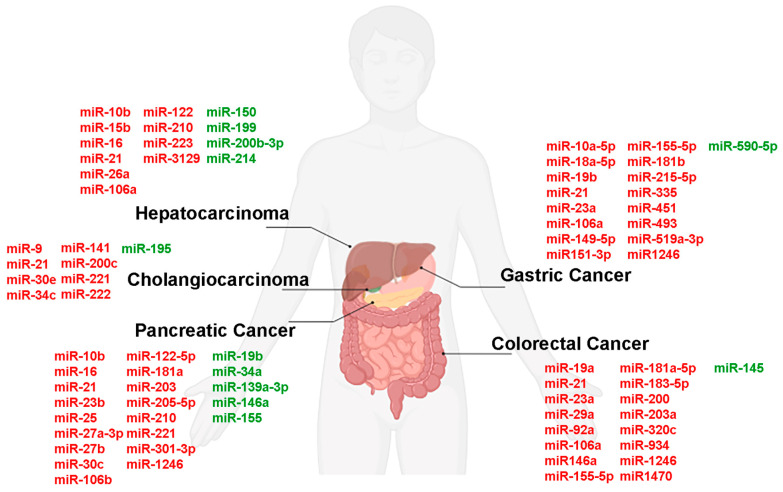
List of EV-derived miRNAs up-(red) and downregulated (green) in GI cancers. Created in BioRender. (https://BioRender.com/p7c6ta2 accessed on 15 December 2025).

Figure 1 is a schematic representation of EV-derived miRNA in GI cancer.

## 4. Conclusions

Traditional biomarkers often fail to provide the necessary sensitivity and specificity for early cancer detection and monitoring therapy response. However, due to their stability in body fluids and their ability to protect, thanks to their lipid bilayer, their molecular cargo from degradation, EVs represent a novel opportunity for non-invasive biomarker assessment, highly suitable for liquid biopsy approaches. Liquid biopsies are less invasive than traditional tissue biopsies and can be repeatedly obtained with minimal discomfort to the patient. This enables continuous monitoring of disease progression and treatment efficacy. EV-associated miRNAs offer several advantages as biomarkers, since they are stable in circulation and protected from RNase degradation. Compared with circulating miRNAs, EV-derived miRNAs offer an improved specificity since EVs are actively released by tumor cells and therefore can reflect distinct cellular states. In addition, surface markers expressed on EVs can enable further isolation and characterization of their cellular origin [182]. Although tissue-derived miRNAs offer a direct and highly specific molecular profile of the tissue of interest, their collection requires biopsy, an invasive and often painful procedure associated with potential clinical risks. Consequently, tissue biopsies are not suitable for routine screening or repeated monitoring.

Furthermore, they can be detected with high sensitivity and specificity using advanced molecular techniques, such as quantitative PCR, microarrays, and next-generation sequencing. These methodological advances have enabled comprehensive profiling of EV-derived miRNAs, facilitating the identification of specific expression signatures linked to tumor type, stage, and even response to therapy. The potential role of EVs in early detection and monitoring of GI cancer is drawing increasing attention. Studies have shown that EVs from GI cancer patients often contain specific tumor-derived proteins, nucleic acids, and other biomolecules that can distinguish them from those of healthy individuals. These tumor-derived EVs can serve as a molecular signature of the underlying neoplasm, providing a rich source of information for early diagnosis and characterization of the disease. The use of liquid biopsy approaches in GI could revolutionize cancer diagnostics, while current imaging and histopathological techniques often fail to capture tumor heterogeneity or detect metastatic disease. In addition to early detection, this novel approach would allow for monitoring of disease progression and evaluation of treatment efficacy with minimal invasiveness, ultimately improving patient survival rates and quality of life. Among the various EV cargoes, EV-associated miRNAs have emerged as particularly interesting biomarker candidates. In the context of GI cancers, EV-associated miRNAs play a crucial role in proliferation, invasion, angiogenesis, and immune evasion. These miRNAs can serve as diagnostic and prognostic markers, and miRNA signatures can also be exploited for patient stratification, driving therapeutic choices and predicting response to specific treatments, including chemotherapy and targeted therapies. Consequently, they may play a key role in the development of personalized therapeutic strategies. In conclusion, the unique biological properties of EVs make them powerful tools for the next generation of cancer diagnostics and monitoring. In the field of GI cancers, where there is an urgent need for non-invasive, accurate, and dynamic biomarkers, EVs, particularly their miRNA cargo, offer a promising approach for improving clinical decision-making and patient outcomes.

## Figures and Tables

**Table 1:** Potential role of EV-derived miRNAs in gastric cancer.

miRNAs	Clinical Application	Biological Role	Sample	References
miR-10a-5p	Diagnostic biomarker	Cell proliferation, metastasis and apoptosis inhibition	Serum	[104]
miR-18a-5p	Early diagnostic biomarker	Cell proliferation and metastasis	Serum	[104,105]
miR-19b	Early diagnostic biomarker	High levels in GC; cancer progression	Serum	[101,104]
miR-21	Prognostic biomarker	Cell proliferation, migration and apoptosis inhibition	Cell culture	[83,99]
miR-23a	Diagnostic biomarker	Promotion of angiogenesis via the repression of PTEN	Cell culture	[96]
miR-106a	Early diagnostic biomarker	Induction of migration and apoptosis reduction via Smad7	Cell culture; Serum	[100,101]
miR-149-5p	Diagnostic and prognostic biomarker	Promotion of GC progression via the AKT1/mTOR pathway	Plasma	[94]
miR-151-3p	Prognostic biomarker	Tumor cell proliferation	Cell culture	[98]
miR-155-5p	Drug resistance	Promotion of chemoresistant phenotype	Cell culture	[95]
miR-181b	Early diagnostic biomarker	High levels in GC; cancer progression	Serum	[105]
miR-215-5p	Diagnostic biomarker	Cell cycle, cell proliferation and metastasis	Serum	[104]
miR-335	Early diagnostic biomarker	High levels in GC; cancer progression	Serum	[105]
miR-451	Prognostic biomarker	Increase in Th17 polarization through exosome-mediated delivery to infiltrating T cells	Serum	[102]
miR-493	Prognostic biomarker	Induction of chemoresistance via MAD2L1 suppression	Peritoneal lavage fluid	[103]
miR-519a-3p	Diagnostic biomarker	Metastasis and angiogenesis	Cell culture	[97]
miR-590-5p	Diagnostic and Prognostic biomarker	High levels in GC; cancer progression	Serum	[106]
miR-1246	Early diagnostic biomarker	High levels in GC; cancer progression	Serum	[107]

**Table 2:** Potential role of EV-derived miRNAs in CRC.

miRNAs	Clinical Application	Biological Role	Sample	References
miR-19a	Diagnostic and prognostic biomarker	Cell proliferation and migration via TIA1	Cancer tissue; Serum	[109,128]
miR-21	Diagnostic and drug resistance biomarker	Induction of cell proliferation, metastasis and chemoresistance by targeting the PTEN/PI3K/AKT	Cell culture; Serum	[114,121,122,123,124,129]
miR-23a	Diagnostic biomarker	Cell proliferation, invasion and metastasis	Serum	[114,122]
miR-29a	Diagnostic biomarker	Upregulated in CRC and associated with disease progression	Serum	[129]
miR-92a	Diagnostic and prognostic biomarker	High levels in stage I-II CRC patients	Serum; Plasma; Cell culture	[113,114,115,125]
miR-106a	Prognostic biomarker	Induction of metastasis through promotion of EMT and TAM2 infiltration	Cell culture	[116]
miR-145	Diagnostic and prognostic biomarker	Suppression of cell migration and invasion by targeting paxillin	Cell culture	[110,111]
miR-146a	Prognostic biomarker	Promotion of tumor metastasis through the CXCL12/CXCR7 axis	Cell culture	[117]
miR-155-5p	Prognostic biomarker	Promotion of tumor metastasis through the CXCL12/CXCR7 axis	Cell culture	[117]
miR-181a-5p	Prognostic biomarker	Promotion of tumor metastasis by activating hepatic stellate cells and remodelling TME	Plasma; Cell culture	[126]
miR-183-5p	Diagnostic and prognostic biomarker	High levels in CRC; cancer progression	Cell culture	[112]
miR-200	Diagnostic biomarker	Promotion of 5-fluorouracil resistant	Cell culture	[130]
miR-203a	Prognostic biomarker	Induction of metastasis by targeting PTEN-induced macrophage polarization	Plasma	[118]
miR-320c	Prognostic and therapeutic biomarker	High levels in CRC; cancer progression	Plasma	[127]
miR-934	Prognostic biomarker	Induction of metastasis through macrophage M2 polarization	Cancer tissue	[119]
miR-1246	Diagnostic biomarker	High levels in CRC; cancer progression	Serum; Plasma	[114,122]
miR-1470	Diagnostic biomarker	Cell proliferation and metastasis	Serum	[131]

**Table 3:** Potential role of EV-derived miRNAs in PC.

miRNAs	Clinical Application	Biological Role	Sample	References
miR-10b	Diagnostic biomarker	High levels in PC; cancer progression	Plasma	[142]
miR-16	Diagnostic biomarker	High levels in PC; cancer progression	Pancreatic juice and serum	[145]
miR-19b	Diagnostic biomarker	High levels in PC; cancer progression	Plasma	[144,146]
miR-21	Diagnostic biomarker	Tumor progression, metastasis, and poor prognosis	Plasma; Pancreatic juice and serum	[142,145]
miR-23b-2p	Diagnostic biomarker	High levels in PC; cancer progression	Plasma; Serum	[143]
miR-25	Diagnostic biomarker	High levels in PC; cancer progression	Pancreatic juice and serum	[145]
miR-27a-3p	Diagnostic biomarker	High levels in PC; cancer progression	Plasma; Serum	[143]
miR-27b	Diagnostic biomarker	High levels in PC; cancer progression	Plasma	[144]
miR-30c	Diagnostic biomarker	Increased cell survival and proliferation	Plasma	[142]
miR-34a	Diagnostic biomarker	Suppression cancer cell proliferation, migration and invasion	Cell culture	[140]
miR-106b	Drug resistance	Induction of gemcitabine resistance	Cell culture	[137]
miR-122-5p	Diagnostic biomarker	High levels in PC; cancer progression	Plasma	[142,144]
miR-146a	Diagnostic and drug resistance biomarker	Suppression of cancer cell invasion	Serum; Cell culture	[135,149]
miR-155	Diagnostic biomarker	Cell proliferation, invasion, and metastasis	Cell culture	[150]
miR-181a	Diagnostic biomarker	High levels in PC; cancer progression	Plasma	[142]
miR-193a-3p	Diagnostic biomarker	Inhibition of cell proliferation and metastasis	Plasma; Serum	[143]
miR-203	Diagnostic and prognostic biomarker	Regulation of tumor cell proliferation via JAK-STAT	Cell culture	[141]
miR-205-5p	Diagnostic biomarker	High levels in PC; cancer progression	Plasma	[144]
miR-210	Drug resistance	Cell invasion and chemoresistance	Cell culture	[73]
miR-221	Diagnostic biomarker	Tumor growth and apoptosis resistance through PTEN and PDCD4	Cell culture	[138]
miR-221-3p	Diagnostic biomarker	High levels in PC; cancer progression	Plasma; Serum	[143,144]
miR-301a-3p	Prognostic biomarker	Promotion of metastasis via the PTEN/PI3Kγ pathway	Cell culture;	[147,148]
miR-1246	Diagnostic biomarker	High levels in PC; cancer progression	Serum; Saliva	[86,135]

**Table 4:** Potential role of EV-derived miRNAs in HCC.

miRNAs	Clinical Application	Biological Role	Sample	References
miR-10b	Diagnostic biomarker	Cell Proliferation and metastasis	Cell culture	[154]
miR-15b	Diagnostic biomarker	High levels in HCC; cancer progression	Serum	[163]
miR-16	Diagnostic biomarker	High levels in HCC; cancer progression	Serum	[163]
miR-21	Diagnostic and prognostic biomarker	High levels in HCC; cancer progression	Cell culture; Serum	[153,154,155,156,159]
miR-26a	Diagnostic biomarker	High levels in HCC; cancer progression	Serum	[163]
miR-106a	Prognostic biomarker	Cell proliferation and invasion via c-Jun pathway	Serum	[164]
miR-122	Diagnostic biomarker	Cell cycle progression, tumor invasion	Serum	[153,160,161];
miR-150	Diagnostic biomarker	High levels in HCC; cancer progression	Serum	[163]
miR-199	Diagnostic biomarker	Inhibition of cell proliferation and migration	Cancer tissues	[153]
miR-200b-3p	Diagnostic biomarker	Inhibition of HCC growth	Cell culture	[158]
miR-210	Diagnostic biomarker	Promotion of angiogenesis	Cell culture	[162]
miR-214	Diagnostic biomarker	Inhibition of cell proliferation	Cell culture	[165,166]
miR-223	Diagnostic biomarker	High levels in HCC; cancer progression	Serum; Plasma	[167,168]
miR-3129	Prognostic biomarker	Promotion of metastasis formation by targeting TXNIP	Plasma	[157]

**Table 5:** Potential role of EV-derived miRNAs in CCA.

miRNAs	Clinical Application	Biological Role	Sample	References
miR-9	Diagnostic biomarker	High levels in CCA; cancer progression	Bile	[173]
miR-21	Diagnostic biomarker	Tumor progression and poor prognosis	Bile; Serum	[172,174]
miR-30e	Diagnosis biomarker	Cell proliferation and invasion	Cell culture	[179,180]
miR-34c	Diagnostic biomarker	Cell growth, invasion, and resistance	Cell culture	[179]
miR-141	Diagnostic biomarker	High levels in CCA; cancer progression	Serum	[177]
miR-195	Diagnostic biomarker	Inhibition of EMT	Cell culture	[181]
miR-200c	Diagnostic biomarker	Regulation of EMT by targeting ZEB1 and ZEB2	Serum	[177,179]
miR-221	Diagnostic biomarker	Cell cycle progression and apoptosis inhibition by targeting p27Kip1 and pro-apoptotic proteins	Cell culture	[176]
miR-222	Diagnostic biomarker	Tumor progression and poor prognosis	Serum	[175]

## Data Availability

No new data were created or analyzed in this study. Data sharing is not applicable to this article.

## Abbreviations

The following abbreviations are used in this manuscript:

Argonaute	(Ago)
Cancer antigen 19-9	(CA19-9)
Cancer-associated fibroblasts	(CAFs)
Carbohydrate antigen 12–5	(CA 12-5)
Carbohydrate antigen 15–3	(CA 15-3)
Carcinoembryonic antigen	(CEA)
Cholangiocarcinoma	(CCA)
Circular RNA	(circRNA)
Circulating cell-free RNA	(cfDNA)
Circulating tumor cells	(CTCs)
Circulating tumor DNA	(ctDNA)
C-Jun N-terminal kinase	(JNK)
Colorectal cancer	(CRC)
Endosomal Sorting Complex Required for Transport	(ESCRT)
Epidermal growth factor receptor	(EGFR)
Epithelial-mesenchymal transition	(EMT)
Extracellular vesicles	(EVs)
Extracellular vesicles derived from gastrointestinal	(GI-Evs)
Factor forkhead box O1	(FOXO1)
Gastric cancer	(GC)
Gastrointestinal	(GI)
Hepatocarcinoma	(HCC)
Heterogeneous nuclear ribonucleoproteins	(hnRNPs)
Intraluminal vesicles	(ILVs)
Log non-coding RNAs	(lncRNAs)
Mammalian target of rapamycin	(mTOR)
Messenger RNAs	(mRNAs)
Microvesicles	(MVs)
mir-34a coated with exosomes	(exomiR-34a)
Mitogen-activated protein kinase	(MAPK)
Pancreatic cancer	(PC)
Pancreatic ductal adenocarcinoma	(PDAC)
Phd finger protein 6	(PHF6)
Piwi-interacting RNA	(piRNA)
Pyruvate dehydrogenase kinase isoform 2	(PDK2)
Regulators of G-protein signaling	(RGS)
RNA-binding proteins	(RBPs)
Small RNAs / microRNAs	(miRNA)
Suppressor of cytokine signaling 1	(SOCS1)
Vascular endothelial growth factor	(VEGF)
Transforming growth factor-beta	(TGF-β)
Tumor microenvironment	(TME)
